# P-701. Adenovirus associated with acute respiratory illness in Korean military trainees: incidence and adenovirus serotypes

**DOI:** 10.1093/ofid/ofae631.897

**Published:** 2025-01-29

**Authors:** Jung Yeon Heo, Soon-Hwan Kwon, Jeyoun Jang, Hong Sang Oh, Young Rong Kim, Hak Jun Hyun, Eun Jin Kim, Young Hwa Choi

**Affiliations:** Ajou University School of Medicine, Suwon-Si, Kyonggi-do, Republic of Korea; Armed Forces Medical Research Institute, Daejeon, Taejon-jikhalsi, Republic of Korea; Armed Forces Medical Research Institute, Daejeon, Taejon-jikhalsi, Republic of Korea; Hallym University Sacred Heart Hospital, Anyang-si, Kyonggi-do, Republic of Korea; Ajou University School of Medicine, Suwon-Si, Kyonggi-do, Republic of Korea; Division of Infectious Diseases, Department of Internal Medicine, Korea University College of Medicine, Seoul, South Korea, Seoul, Seoul-t'ukpyolsi, Republic of Korea; Ajou University School of Medicine, Suwon-Si, Kyonggi-do, Republic of Korea; Ajou University School of Medicine, Suwon-Si, Kyonggi-do, Republic of Korea

## Abstract

**Background:**

Previous studies suggested that that human adenovirus (HAdV) is the most prevalent in patients with acute lower respiratory tract infection and is the most common cause of pneumonia in Korean military. A sentinel surveillance program was initiated to investigate the incidence of acute respiratory illness (ARI) and characteristics of type-specific HAdV respiratory infections in Korean military trainees.

Weakly incidence of acute respiratory infection per 1,000 persons in military trainees

**Figure:**
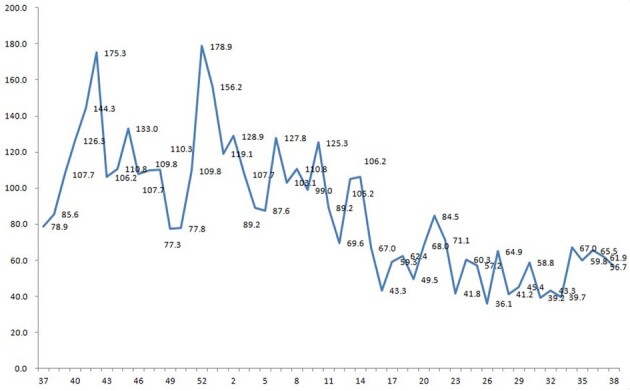

**Methods:**

To calculate the incidence of ARI, all new trainees with febrile respiratory illness (FRI) who were required to visit the infirmaries at three military training centers were enrolled from September 2016 to August 2017. Of soldiers with FRI, 10 nasopharyngeal or throat swab specimens were weakly collected for for HAdV molecular typing.

Weakly proportion of adenovirus infection in trainees with acute respiratory infection

**Figure:**
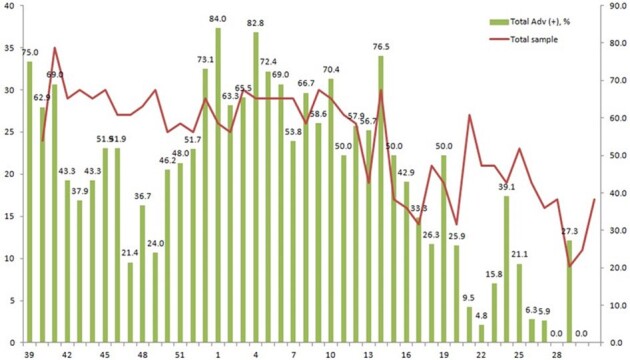

**Results:**

The incidence of ARI, which was defined as the number of weakly ARI cases per 1,000 trainees, was estimated at 7.5-375.5 cases per 1,000 trainees with peaks between 41th week and 10th week in 2016-17 season. Of 1,079 patients with FRI, HAdVs were detected in 518 (48.0%) patients. HAdV type 55 was the most prevalent virus (98.6%, 511 cases). HAdV type 2 was only identified in 7 cases. Although HAdV associated ARI occurred throughout the year, Patients with HAdV infection were primarily observed during winter and spring. The clinical diagnoses in patients with HAdV associated ARI were almost pharyngotonsillitis (96.3%). Pneumonia (2.2%) was uncommon manifestation. There was no statistically significant difference in demographic characteristics and presenting symptoms and signs between HAdV-positive and HAdV-negative patients.

**Conclusion:**

HAdV-55 was the most prevalent type in Korean soldiers with HAdV-associated ARI. Adenovirus type 55 was revealed to be the most important causative agent of acute respiratory infection in military trainees. Further studies are required to verify which HAdV types are associated with ARI in military recruits.

**Disclosures:**

**All Authors**: No reported disclosures

